# Effects of the Crystalline Properties of Hollow Ceria Nanostructures on a CuO-CeO_2_ Catalyst in CO Oxidation

**DOI:** 10.3390/ma15113859

**Published:** 2022-05-28

**Authors:** Se-Jin Jang, Hyeonkyeong Lee, Jiyull Kim, Na-Yeon Kim, Dong-Seop Choi, Ji Bong Joo

**Affiliations:** Department of Chemical Engineering, Konkuk University, 120 Neungdong-ro, Gwangjin-gu, Seoul 05029, Korea; rkddnr1205@konkuk.ac.kr (S.-J.J.); hyeonk@konkuk.ac.kr (H.L.); jiyull0630@konkuk.ac.kr (J.K.); kny960403@konkuk.ac.kr (N.-Y.K.); cds1105@konkuk.ac.kr (D.-S.C.)

**Keywords:** hollow CeO_2_, crystallinity control, CuO-CeO_2_, CO oxidation

## Abstract

The development of an efficient and economic catalyst with high catalytic performance is always challenging. In this study, we report the synthesis of hollow CeO_2_ nanostructures and the crystallinity control of a CeO_2_ layer used as a support material for a CuO-CeO_2_ catalyst in CO oxidation. The hollow CeO_2_ nanostructures were synthesized using a simple hydrothermal method. The crystallinity of the hollow CeO_2_ shell layer was controlled through thermal treatment at various temperatures. The crystallinity of hollow CeO_2_ was enhanced by increasing the calcination temperature, but both porosity and surface area decreased, showing an opposite trend to that of crystallinity. The crystallinity of hollow CeO_2_ significantly influenced both the characteristics and the catalytic performance of the corresponding hollow CuO-CeO_2_ (H-Cu-CeO_2_) catalysts. The degree of oxygen vacancy significantly decreased with the calcination temperature. H-Cu-CeO_2_ (HT), which presented the lowest CeO_2_ crystallinity, not only had a high degree of oxygen vacancy but also showed well-dispersed CuO species, while H-Cu-CeO_2_ (800), with well-developed crystallinity, showed low CuO dispersion. The H-Cu-CeO_2_ (HT) catalyst exhibited significantly enhanced catalytic activity and stability. In this study, we systemically analyzed the characteristics and catalyst performance of hollow CeO_2_ samples and the corresponding hollow CuO-CeO_2_ catalysts.

## 1. Introduction

Carbon monoxide (CO) is a colorless, odorless, tasteless, and flammable gas that is generally released by incomplete combustion reactions of hydrocarbon chemicals. It is one of the most useful chemicals in the petrochemical industry. The syngas, which mainly consist of CO and hydrogen, has great potential to produce many useful platform chemicals [1,2,3,4]. It is well known that CO/H_2_ can be converted to methanol using a Cu-based catalyst under pressured conditions and can produce gasoline by the Fischer–Tropsch process using iron-based catalysts [5,6,7,8]. However, CO plays a role as an anthropogenic toxic pollutant when it is released at levels beyond the those established by environmental regulations. It readily combines with hemoglobin in the blood to produce carboxyhemoglobin, which significantly hampers oxygen delivery. It is well known that concentrations as low as 667 ppm might cause up to 50% of the body’s hemoglobin to convert to carboxyhemoglobin, resulting in seizure, coma, and fatality. Therefore, harmful CO from incomplete combustion conditions must be removed for not only protection from secondary damages but also health issues.

There are several methods for removing toxic CO that have been evaluated in both fundamental and practical engineering studies. The major approaches are based on adsorption, thermal combustion, and catalytic oxidation [9,10,11,12,13,14]. Although adsorption is the simplest and most extensively studied method for CO removal, adsorption saturation is easily achieved, as the adsorption column becomes larger since conventional adsorbents have a low affinity and small adsorption capability toward CO [9,10]. Thermal combustion can allow CO to be oxidized to CO_2_. However, an additional combustion burner must be installed, which operates in high-temperature environments, resulting in high energy costs. Catalytic CO oxidation is one of the most favorable methods to convert CO to CO_2_. Heterogeneous catalysts allow CO to be oxidized in a considerably low-temperature range of 50–150 °C [15,16,17,18]. In addition, they show high efficiency and a variety of applications, which has fostered progress in both fundamental lab-scale research and practical industrial applications.

Currently, numerous catalysts have been investigated in CO oxidation. The catalysts based on noble metals such as Pt, Pd, Rh, and Au exhibited excellent performance at low-temperatures [19,20,21,22]. The Au nanoparticle-supported TiO_2_ catalyst, in particular, showed extremely outstanding activity even at room temperature [23]. However, the use of these noble metal catalysts in large-scale reactors is usually difficult due to high cost and limited resources, which poses a concern in practical applications. In addition, noble metal nanoparticles on the support surface are sometimes sintered, becoming large under relatively high temperatures, resulting in the loss of the outstanding activity that was observed in small nanoparticles [24]. Non-precious transition metals such as Cu and Ni have been used as alternative resources. CuO-based catalysts have been intensively investigated in both fundamental research and practical CO removal processes [13,14,18]. These CuO-based catalysts, with wide availability, low cost, and high activity, have been confirmed as some of the most effective catalysts in CO oxidation as well as in other oxidation processes.

Various metal oxides such as SiO_2_, TiO_2_, Fe_2_O_3_, CeO_2_, etc., have been used as support materials for CO oxidation [15,25,26,27,28,29,30]. Among the various oxide support materials, metal oxides having high oxygen storage capability, oxygen mobility and redox properties have been widely known for their ability to significantly improve the catalytic performance of the supported metal catalysts in CO oxidation reactions [30]. Cerium oxide (CeO_2_) is one of the representative oxide materials, which has two oxidation states—trivalent and tetravalent—and redox characteristics [29,30,31]. The ability of ceria to undergo rapid redox cycles is responsible for its oxygen storage capacity (OSC). It is widely known that nanostructured CeO_2_ particles having small crystalline grains present improved redox properties, higher ionic conductivity, and better catalytic activity than micro-sized particles with relatively large crystalline grain. It should be noted that the loss of oxygen storage capacity due to the sintering of CeO_2_ particles and grain growth at high temperatures results in a relatively low catalytic performance. 

Currently, various studies have investigated the catalytic performance enhancement following the application of nanostructured CeO_2_ as a support material. May et al. prepared (100) plane-oriented CeO_2_ nanocubes and deposited 1% Cu on the surface of the CeO_2_ nanocubes. They reported that this system achieved an improved catalytic performance due to the increased reduction of the supported copper species resulting from weak metal–support interactions [32]. Maciel et al. prepared a CeO_2_ support by hydrothermal synthesis and precipitation. They deposited 5 wt.% Cu species on each CeO_2_ support and carried out a preferential CO oxidation (PROX) reaction. They reported that the Cu/CeO_2_ sample prepared by hydrothermal synthesis with a small CeO_2_ crystal size showed higher performance [33]. It can be concluded that ceria with a small crystallite size has enhanced redox capability and the more reactive copper species are highly dispersed on CeO_2_, resulting in high performance in the PROX reaction.

It is also widely known that hollow nanostructures show many advantageous characteristics in catalytic reactions [34,35,36,37,38]. Hollow nanostructures with a porous shell layer possess a large surface area per unit mass. The mesoporous shell layer not only provides a short diffusion pathway but also helps the reactant molecules to easily access the active sites. In addition, molecule diffusion followed by surface reaction can proceed easily without any stagnation, since most of the solid core positions of the submicron particles are empty. Practically, hollow nanostructured catalysts showed outstanding performance in many catalytic reactions. Hollow TiO_2_ nanostructures have been successfully applied as photocatalysts for photocatalytic organic decomposition and hydrogen production [39,40,41]. In addition, it is well known that hollow sulfated ZrO_2_ exhibited better performance in dehydration reaction than its solid counterpart [37]. Other shell-based nanostructures have also been neatly synthesized and successfully used in catalytic applications. Metal@graphene-type core-shell nanostructures are finely synthesized and used as catalysts in the selective oxidation of alcohol in liquid phase [42,43]. Based on previous studies and our hypothesis, it is believed that metal-supported hollow CeO_2_ with small crystalline grains can be an efficient catalyst for gas-phase oxidation reactions. 

So far, there have been several studies on the synthesis of hollow CeO_2_ spheres for catalysis applications. Most of the works focused on the novel synthesis of hollow CeO_2_ materials. The systemic study of the relationship between the physiochemical properties and the catalytic performance enhancement of hollow CeO2-based catalysts is necessary. In our work, we tried to elucidate the essential features that can improve the catalytic properties of hollow CeO_2_ support materials and intentionally control them to improve the catalytic performance of hollow CeO_2_-based catalysts. In this study, we tried to characterize the relationship between crystallinity, degree of oxygen vacancy, and catalysis performance enhancement. Based on the above-reported knowledge, we devoted our efforts to proposing an optimal catalyst for low-temperature CO oxidation.

We synthesized a hollow CeO_2_ nanostructure that was used as a support material for a CuO-supported catalyst for CO oxidation. The hollow CeO_2_ particles were synthesized through modified hydrothermal synthesis followed by calcination. The crystallinity of the hollow CeO_2_ samples was controlled as the calcination temperature increased. When CuO was deposited on H-CeO_2_ (HT), which has the highest surface area and the smallest crystal grain, the resulting H-Cu-CeO_2_ (HT) catalyst exhibited high active metal dispersion, favorable surface oxygen transfer characteristics, and excellent catalytic performance in low-temperature CO oxidation. In this study, we discuss the crystalline characteristics of hollow CeO_2_ samples and the catalytic performance of the corresponding CuO-CeO_2_ catalysts.

## 2. Materials and Methods

### 2.1. Materials 

Cerium (III) nitrate hexahydrate (Ce(NO_3_)_3_∙6H_2_O, 98%) and Copper (II) nitrate trihydrate (Cu(NO_3_)_2_∙3H_2_O, 99%) were purchased from Samchun Chemical Company (Seoul, Korea). Acetic acid (CH_3_COOH) and ethylene glycol (EG, C_2_H_4_(OH)_2_) were obtained from Daejung Chemical Company (Gyeonggi-Do, Korea). D.I. water and ethanol were used throughout the experiments, and all chemicals were used as received.

### 2.2. Synthesis 

Hollow CeO_2_ nanostructures were synthesized through a modified hydrothermal method previously reported [44,45]. Ce(NO_3_)_3_∙6H_2_O (2 g) was dissolved into EG (80 mL) under vigorous stirring for 2 h. Subsequently, D.I. water (6 mL) and acetic acid (2 mL) were added to the above mixture and stirred for 1 h for homogeneous mixing. The above solution was then transferred into a Teflon-lined autoclave and heated at 190 °C for 9 h. The precipitate was isolated by centrifugation and washed with ethanol 3 times. The solid sample was then dried at 80 °C for 12 h. The dried sample showed a hollow morphology; the as-synthesized hollow CeO_2_ was denoted as H-CeO_2_ (HT). The term H-CeO_2_ (HT) indicates the hollow-CeO_2_ sample as-synthesized right after the hydrothermal (HT) step. HT indicates the hydrothermal (HT) synthetic step.

To control the crystallinity of hollow CeO_2_, the H-CeO_2_ (HT) sample was subjected to additional heat treatment. The dried H-CeO_2_ (HT) sample was placed in an alumina boat in a muffle furnace and calcined at the desired temperatures (300, 500, and 800 °C) for 2 h under air conditions. The calcined hollow CeO_2_ samples were termed H-CeO_2_ (X) (where X is the calcination temperature).

H-CeO_2_-supported Cu catalysts (H-Cu-CeO_2_) were synthesized through the wet impregnation method. A Cu precursor (Cu(NO_3_)_2_.3H_2_O) solution was impregnated into the calcined H-CeO_2_ (x) supports, and the resulting mixture was dried at 80 °C for 12 h. The dried Cu-impregnated H-CeO_2_ (x) samples were then calcined at 500 °C for 2 h under air conditions to produce the final H-Cu-CeO_2_ (x) catalysts. During the synthesis of the H-Cu-CeO_2_ catalysts, we tried to load 11 wt.% CuO on the pre-synthesized H-CeO_2_ supports.

### 2.3. Characterizations 

Particle morphology and dimension were investigated using scanning electron microscopy (SEM, JSM-6060, JEOL, Tokyo, Janpan) and transmission electron microscopy (TEM, JEM-2100, JEOL). The crystalline properties of the samples were determined through X-ray diffraction (XRD) analysis using a Rigaku D/mas-2200 diffractometer with Cu Kα radiation (λ = 1.5406 Å). The grain size of the crystallite can be calculated with the Scherrer equation [18]:

$$D=\frac{K\lambda }{\beta cos\theta }$$

where *K* represents the Scherrer constant (0.9), λ represents the X-ray wavelength (λ = 1.5406 Å), *β* represents the line broadening at half the maximum intensity (FWHM) in radians, and *θ* represents the Bragg angle. N_2_ adsorption isotherms were obtained at 77 K using a nitrogen sorption instrument (TriStar II 3020, Micrometics, Norcross, GA, USA). Pore size distributions were estimated using the Barrett–Joyner–Halenda (BJH) formula from the adsorption branches of N_2_ isotherms. Raman spectra were obtained using a spectrophotometer (SR-303i, Andor Technology, Belfast, UK) with a 532 nm laser module. The hydrogen temperature-programmed reduction (H_2_-TPR) measurement was carried out using a conventional temperature-programmed instrument (BELCAT-M, MicrotracBEL Corp, Osaka, Japan). The catalyst (50 mg) was pretreated in He for 30 min at 200 °C before the TPR measurement to remove any impurities, then cooled to room temperature. H_2_ balanced with Ar gas (5%, 30 mL/min) was introduced to the catalyst, and the catalyst bed was programmatically heated to 800 °C at a heating rate of 5 °C/min. The consumption of hydrogen was monitored by a thermal conductivity detector (TCD). The chemical composition and relative amount of CuO on each final H-Cu-CeO_2_ catalyst were determined by X-ray Fluorescence analysis (XRF, Epsilon 3-XL, PANaytical, Worcestershire, UK). As shown in Table S1, all H-Cu-CeO_2_ catalysts showed values close to 11 wt.%, indicating that the metal was finely loaded as planned.

### 2.4. CO Oxidation 

Catalytic CO oxidation was carried out using a homemade fixed-bed reaction system in the temperature range of 40 to 200 °C. The catalyst (50 mg) was placed in the fixed bed, and the bed temperature was monitored by a K-type thermocouple placed right above the bed. The reaction temperature was controlled by using a PID temperature controller. The flow rates of gases (CO, N_2_, O_2_) were controlled by a mass flow controller (MFC). The bed temperature was controlled at 40 °C before the catalytic activity test. Once the bed temperature was stable, the feed gas was introduced to the reactor with the gas composition of CO:O_2_:N_2_ = 0.01:0.2:0.79 at a space velocity of 60,000 mL/h∙g_cat_. After 30 min, the bed temperature was continuously increased at a rising rate of 1 °C/min to 200 °C, and the effluent gases were analyzed by a gas chromatographer (GC, Agilent 6890) equipped with a thermal conductivity detector (TCD). Carboxen-1000 (SUPELCO Analytical, Bellefonte, PA, USA) was used as a GC column, and He was used as a carrier gas to quantify CO, O_2_, and N_2_ in the effluent gas. The following equation was used to calculate CO conversion:

$$\mathrm{CO}\;\mathrm{conversion}\;\left(\%\right)=\frac{f_{\left[\mathrm{CO}\right]in}-f_{\left[\mathrm{CO}\right]out}}{f_{\left[\mathrm{CO}\right]in}}\times 100$$

where *f* represents the molar fraction of each component. 

The activation energy, which is one of the important kinetic parameters in the CO oxidation reaction, was calculated at a low temperature between 60 °C and 90 °C, using the following Arrhenius equation:

$$\ln r=-\frac{E_{a}}{RT}+\ln A$$

where *r* represents the reaction rate of the CO oxidation reaction, *E_a_* represents the activation energy (J mol^−1^), *R* represents the gas constant (J mol^−1^ K^−1^), and *T* represents the reaction temperature (K). The reaction rate was calculated using the Equation below:

$$r_{co}=\frac{F_{\mathrm{co}}X_{\mathrm{co}}}{m_{cat}}$$

where *F_CO_* represents the flow rate (mol/s), *X_CO_* represents the conversion of CO, *m_cat_* represents the catalyst weight (g), and *r_co_* represents the reaction rate (mol/g•s).

## 3. Results and Discussion

**Figure 1:**
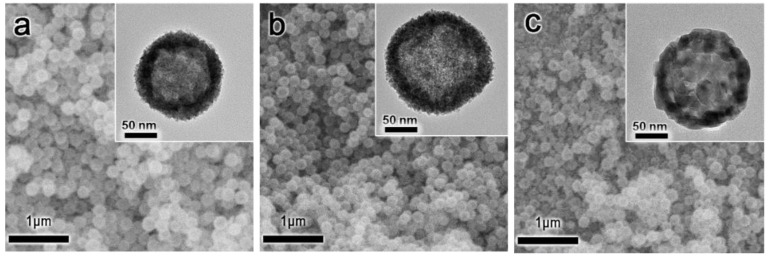
SEM and TEM (inset) images of the H-CeO_2_ samples: (**a**) H-CeO_2_ (HT), (**b**) H-CeO_2_ (500), and (**c**) H-CeO_2_ (800).

The morphologies of the as-synthesized H-CeO_2_ and the calcined H-CeO_2_ samples were investigated using both SEM and TEM. Figure 1a shows the H-CeO_2_ (HT) sample which revealed a uniform spherical morphology with a diameter of ca. ~180 nm. In Figure 1a inset, it is easily observed that the H-CeO_2_ (HT) sample showed an empty core portion with a porous shell layer having a small CeO_2_ crystallite grain of ca. 5–6 nm. When the calcination temperature was increased to 500 and 800 °C, the spherical morphology was well maintained, indicating thermal stability of the synthesized H-CeO_2_ particles. The average diameters of H-CeO_2_ (500) and H-CeO_2_ (800) were ca. ~180 and ~175 nm, almost similar to that of the original H-CeO_2_ (HT) (Figure 1b,c). As shown in inset images of Figure 1b,c, both H-CeO_2_ (500) and H-CeO_2_ (800) samples showed a obvious hollow morphology. The CeO_2_ crystallite grains in the shell layer continuously became larger as the calcination temperature increased. The observed crystallite grain of H-CeO_2_ (500) and H-CeO_2_ (800) were ca. 8 and 25 nm in size, respectively. This indicated that the crystallinity of CeO_2_ increased as the calcination temperature increased. Although the H-CeO_2_ (800) sample showed a little portion of a broken fragment of CeO_2_ due to severe thermal crystallization, the majority of the particles showed the spherical morphology, indicating a well-maintained structural integrity [29]. We also investigated the morphology of the H-Cu-CeO_2_ catalysts using SEM and TEM (Figure S1). After Cu loading followed by calcination under air, the structural integrity of the resulting H-Cu-CeO_2_ catalysts was well maintained. After the calcination steps, the Cu species were converted to CuO species. Since both the CuO particles and the hollow CeO2 support consisted of metal oxide materials, the contrast difference was small in TEM analysis; therefore, no obvious CuO particles were observed on the CeO_2_ support surface (Figure S1 inset). The catalysts showed a similar structural morphology compared to mother H-CeO_2_. They could be used as catalysts in CO oxidation, later discussed.

**Figure 2:**
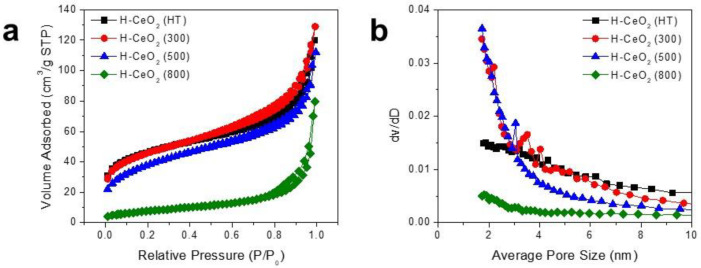
(**a**) Nitrogen isotherms and (**b**) BJH pore size distributions of the H-CeO_2_ samples.

We investigated the pore characteristics of the H-CeO_2_ samples. Figure 2a shows the nitrogen isotherms and corresponding BJH pore size distributions of the H-CeO_2_ samples. The as-synthesized H-CeO_2_ (HT) sample showed the main adsorption at a low relative pressure (0–0.1 P/P_0_) and continuous adsorption in the range of 0.1–0.7 P/P_0_. The type II isotherm indicated a porous CeO_2_ shell structure consisting of both micropores and mesopores. The H-CeO_2_ (300) sample calcined at a relatively low temperature (300 °C) and showed a similar isotherm pattern, indicating the presence of both micropores and mesopores in the CeO_2_ shell. The H-CeO_2_ (HT) and H-CeO_2_ (300) samples had a large adsorption capacity in the range of monolayer adsorption (0.1 < P/P_0_ < 0.25), which indicated a relatively large BET surface area with a porous structure. The amount of monolayer adsorption continuously decreased when calcination was carried out at relatively high temperatures (500 and 800 °C). The amount of monolayer adsorption of the H-CeO_2_ samples followed the order: H-CeO_2_ (HT) > H-CeO_2_ (300) > H-CeO_2_ (500) >> H-CeO_2_ (800). The measured surface area for H-CeO_2_ (HT), H-CeO_2_ (300), H-CeO_2_ (500), and H-CeO_2_ (800) was 155.5, 155.8, 133.9, and 29.0 m^2^/g, respectively. 

Figure 2b shows the pore size distributions of the H-CeO_2_ samples. All the H-CeO_2_ samples showed both micropores and mesopores with a size in the range of 1–8 nm. H-CeO_2_ (HT) and H-CeO_2_ (300) showed a larger dV/dD value in the range of 3–8 nm. As the calcination temperature increased, the H-CeO_2_ (500) sample exhibited a decreased dV/dD value in the pore size range of 3–8 nm, indicating that the crystallinity of the hollow CeO_2_ layer increased resulting in the shrinkage of a major portion of mesopores. The H-CeO_2_ (800) sample did not exhibit any obvious distribution peak in the size range of mesopores when calcination was carried out at 800 °C, indicating an almost non-porous structure in the CeO_2_ shell layer. 

In addition, we also investigated the N_2_ isotherms of the H-Cu-CeO_2_ catalysts. As shown in Figure S2, the H-Cu-CeO_2_ catalysts showed a slightly smaller adsorption capacity in the range of monolayer adsorption (0.1 < P/P_0_ < 0.25) than the mother H-CeO_2_ support, indicating that the specific surface area was decreased by CuO loading followed by calcination at 500 °C. The specific surface area values of H-Cu-CeO_2_ (HT), H-Cu-CeO_2_ (300), H-Cu-CeO_2_ (500), and H-Cu-CeO_2_ (800) were 102.0, 105.6, 100.9, and 21.03 m^2^/g, respectively. In addition, even though the H-Cu-CeO_2_ catalysts showed a slightly smaller adsorption capacity, they showed N_2_ isotherm patterns similar to those of mother H-CeO_2_ supports, indicating that the H-Cu-CeO_2_ catalysts have similar pore structures, with micropores and mesopores in the CeO_2_ shell.

**Figure 3:**
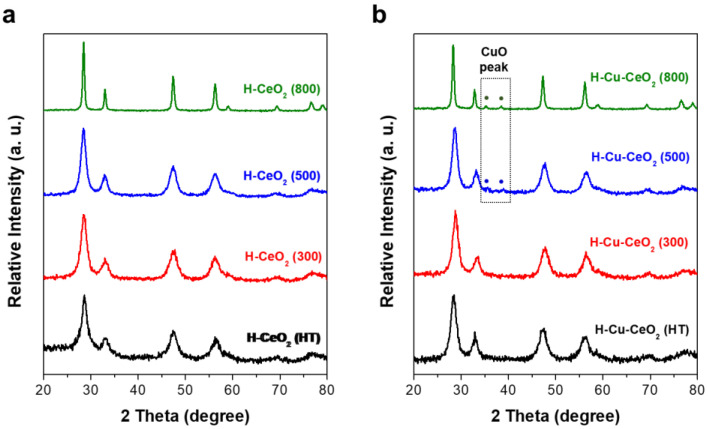
XRD pattern of (**a**) the H-CeO_2_ support and (**b**) the H-Cu-CeO_2_ catalysts.

The crystalline characteristics of H-CeO_2_ and corresponding H-Cu-CeO_2_ catalyst were investigated using X-ray diffraction (XRD). As shown in Figure 3a, H-CeO_2_ (HT) showed the typical diffraction peaks of a face-centered cubic (fcc) fluorite structure of ceria at 2*θ* = 28.6°, 33.1°, 47.6°, 69.34°, and 76.5°, that were attributed to the (111), (200), (220), (311), and (331) planes, respectively. As the calcination temperature increased, the dominant CeO_2_ peaks became even sharper, indicating that the CeO_2_ crystal grains became larger because of thermal growth. Although the dominant CeO_2_ peaks continuously enlarged as the calcination temperature increased, no obvious changes in the diffraction peaks were observed., indicating that the fcc crystalline structure of CeO_2_ was well maintained even at high temperatures. The average crystalline sizes of the samples were calculated using the Scherrer formula. The average anatase grain sizes were determined to be approximately 5.5, 5.9, 7.4, and 17.3 nm for H-CeO_2_ (HT), H-CeO_2_ (300), H-CeO_2_ (500), and H-CeO_2_ (800), respectively.

The crystalline properties of the H-Cu-CeO_2_ catalysts were also investigated after Cu impregnation followed by calcination. As shown in Figure 3b, H-Cu-CeO_2_ (HT) and H-Cu-CeO_2_ (300) showed diffraction peaks related to fcc CeO_2_, which were identical to the peaks observed for H-CeO_2_ (HT) and H-CeO_2_ (300), as shown in Figure 3a, indicating that the fcc CeO_2_ crystalline structure was well maintained during Cu impregnation followed by thermal calcination at 500 °C. There was no obvious peak related to CuO or Cu species, indicating that not only that tiny CuO species were highly dispersed within the CeO_2_ shell layer but also that the content of the CuO particles was below the detection limit of XRD. When Cu was impregnated on H-CeO_2_ (500) followed by calcination, small diffraction changes were observed at ca. 34.5 and 48.5°, which contributed to the (002) and (111) planes of CuO. The H-Cu-CeO2 (800) sample showed even more obvious diffraction peaks in the same positions (Figure S3) [34]. Therefore, it can be concluded that variation in the dispersity of CuO can be explained by the intrinsic characteristics of each hollow CeO_2_ support, since the same amount of Cu was impregnated on different supports and calcined at the same temperature. As discussed in a later section, H-CeO_2_ (HT) showed a higher surface area and could have a higher amount of oxygen vacancy compared to H-CeO_2_ (500) and H-CeO_2_ (800). Thus, H-CeO_2_ (HT) can provide a relatively larger surface for CuO dispersion, and its oxygen vacancies could provide anchoring sites for the formation of either isolated CuO species or Cu-O-Ce species. However, the H-CeO_2_ (500) and H-CeO_2_ (800) samples allowed a more limited dispersion of the CuO species, which led to the formation of relatively larger CuO nanoparticles. 

**Figure 4:**
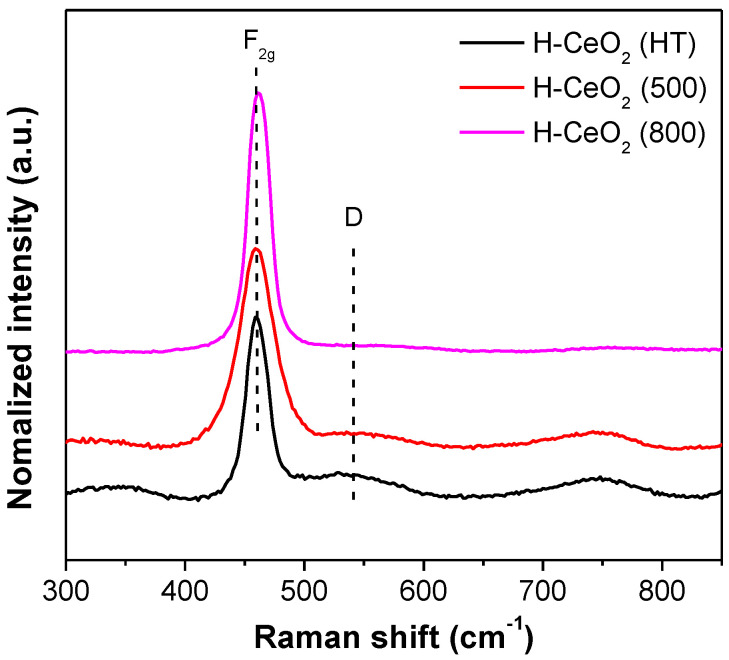
Raman spectra of H-CeO_2_ supports.

Raman spectra were also obtained to confirm the surface structural properties of the H-CeO_2_ samples. As shown in Figure 4, there strong Raman peaks were observed at 458 cm^−1^ due to the F_2g_ Raman active mode of the fcc fluorite structure of CeO_2_ materials, indicating a symmetric breathing mode of oxygen atoms near the Ce^4+^ ions. In addition, we observed weak shoulders in the 480–600 cm^−1^ range, indicating the degree of oxygen vacancies caused by the presence of Ce^3+^ ions [46]. We compared the relative area ratio of A(D) to A(F_2g_) in the Raman curves to determine the degree of both oxygen vacancies and defects on each H-CeO_2_ sample. The relative area ratio of the H-CeO_2_ samples followed the order: H-CeO_2_ (HT) > H-CeO_2_ (500) > H-CeO_2_ (800). Based on the above results, it should be noted that the degree of oxygen vacancy in the ceria support depended on CeO_2_ crystallinity, which is mainly controlled by calcination [46]. It is widely known that a large degree of oxygen vacancy and defects has a positive impact during catalysis, resulting in high activity in oxidation reactions [29,32]. In our study, the degree of oxygen vacancy of H-CeO_2_ was tuned by controlling crystallinity through thermal calcination. The H-CeO_2_ (HT) sample showed limited developed crystallinity, a large surface area, and a high degree of oxygen vacancy. The high degree of oxygen vacancy may help Cu active sites of the resulting H-Cu-CeO_2_ catalysts to activate oxygen and to accelerate surface oxygen mobility in CO oxidation, resulting in high performance.

XPS quantification were carried out to analyze the chemical state of both H-CeO_2_ (x) supports and H-Cu-CeO_2_ (x) catalysts. Figure S4a indicates high-resolution Ce 3d spectra with peak deconvolution of H-CeO_2_ (HT), H-CeO_2_ (800), H-Cu-CeO_2_ (HT), and H-Cu-CeO_2_ (800). Since the Ce atom in ceria has several chemical states, the spectra can be deconvoluted to several peaks. The spectra of the H-CeO_2_ and H-Cu-CeO_2_ samples were deconvoluted into 10 peaks due to Ce 3d_5/2_ (labelled as v) and Ce 3d_3/2_ (labelled as u) contributions. The *v^0^* and *v’* peaks were attributed to Ce^3+^ states, while the *v, v”,* and *v”’* bands were attributed to Ce^4+^ states. Similarly, the *u^0^,* and *u’* peaks were attributed to Ce^3+^, and the *u, u”,* and *u”’* peaks indicated Ce^4+^ states [47,48]. It is well known that the existence of Ce^3+^ in ceria is associated with the formation of oxygen vacancies, and a higher concentration of Ce^3+^ implies larger amounts of oxygen vacancies [49,50]. After deconvoluting all XPS spectra, we estimated the relative Ce^3+^ ratio of both H-CeO_2_ (x) supports and H-Cu-CeO_2_ (x) catalysts by the following relationship:

relative Ce^3+^ ratio = area of Ce^3+^/area of (Ce^4+^ + Ce^3+^)

The calculated Ce^3+^ area ratios of H-CeO_2_ (HT), H-CeO_2_ (800), H-Cu-CeO_2_ (HT), and H-Cu-CeO_2_ (800) were ca. 0.59, 0.38, 0.35, and 0.23, respectively. This indicated H-CeO_2_ (HT) and H-Cu-CeO_2_ had a much higher number of oxygen vacancies than H-CeO_2_ (800) and H-Cu-CeO_2_ (800). This result is consistent with the Raman analysis.

Figure S4b indicates high-resolution Cu 2p spectra with peak deconvolution of H-Cu-CeO_2_ (HT) and H-Cu-CeO_2_ (800) catalysts. The Cu 2p spectra of both H-Cu-CeO_2_ (HT) and H-Cu-CeO_2_ (800) exhibited a principal peak which consisted of two deconvoluted peaks at 931 eV and 933 eV, ascribed to a mixed state consisting of Cu^+^ and Cu^2+^ [34]. The peak related to the Cu^+^ state indicated the formation of a Cu-O-Ce bond, while the Cu^2+^ state should be closely related to bulk CuO. After deconvoluting all XPS spectra, we estimated the relative Cu^+^ ratio of both H-Cu-CeO_2_ (x) catalysts by the following relationship:

relative Cu^+^ ratio = area of Cu^+^/area of (Cu^+^ + Cu^2+^)

The calculated Cu^+^ ratios of H-Cu-CeO_2_ (HT) and H-Cu-CeO_2_ (800) were 0.28 and 0.23, respectively. It indicated that H-Cu-CeO_2_ (HT) contained highly dispersed Cu species rather than bulk CuO particles compared to H-Cu-CeO_2_ (800), consistent with the XRD results [34]. In addition, it was easily observed that both H-Cu-CeO_2_ (HT) and H-Cu-CeO2 (800) showed the obvious satellite peaks. The reductive characteristics of the Cu species can be compared to the ratio of the intensity of the satellite peak to the intensity of the principal peak (I_sat_/I_pp_) [51]. The calculated I_sat_/I_pp_ values for H-Cu-CeO_2_ (HT) and H-Cu-CeO_2_ (800) were 0.35 and 0.18, respectively. It means that H-Cu-CeO_2_ (HT) showed more reductive Cu species than H-Cu-CeO_2_ (800), consistent with the TPR results, as discussed later. 

**Figure 5:**
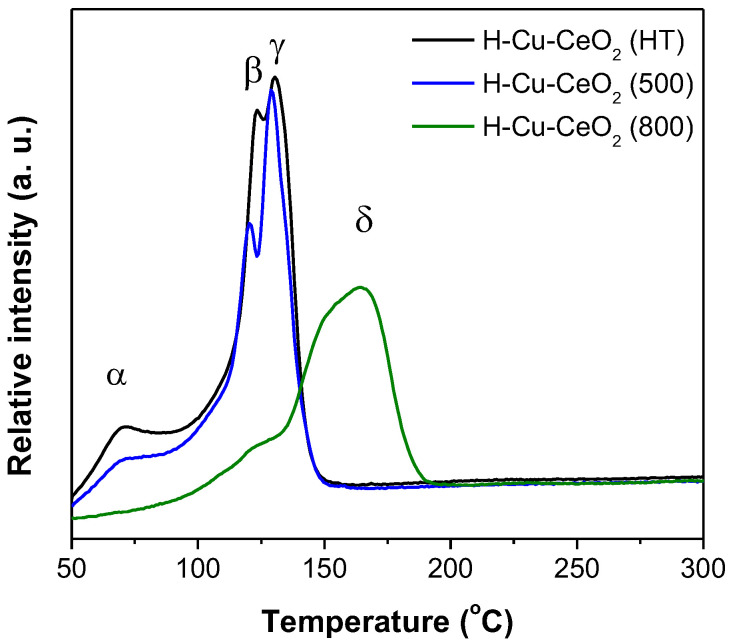
H_2_-TPR profiles of the H-Cu-CeO_2_ catalysts.

To identify the Cu species and the metal–support interactions on H-CeO_2_ supports having different crystallinity, the H_2_-TPR profiles of the H-Cu-CeO_2_ catalysts were investigated. Figure 5 shows the H_2_-TPR profiles of the H-Cu-CeO_2_ catalysts. There were four types of copper-oxygen species over the supported copper oxide catalysts [30]: (i) isolated copper oxide species, which could interact with the corresponding support closely (α), (ii) weak magnetic associates including several Cu^2+^ ions, which were in close contact with each other (β), (iii) small two- or three-dimensional CuO clusters (γ), and (iv) crystalline CuO, with properties and characteristics similar to those of pure CuO powders (δ). The H-Cu-CeO_2_ (HT) and H-Cu-CeO_2_ (500) catalysts showed α, β, and γ reduction peaks at ca. 70, 125, and 140 °C, respectively, in the TPR profiles. H-Cu-CeO_2_ (HT) showed larger α and β peaks than H-Cu-CeO_2_ (500), indicating that it had a large portion of isolated copper oxide species and Cu magnetic associates including several Cu^2+^ ions. Based on the H_2_-TPR results, it should be noted that the H-Cu-CeO_2_ (HT) catalyst had highly dispersed Cu species (isolated copper oxide species and magnetic associates including several Cu^2+^ ions) which closely interacted on the surface of the H-CeO_2_ support [30,31]. It also presented well-dispersed CuO clusters. In addition, the H-Cu-CeO_2_ (HT) sample should have a Cu-O-Ce bonding structure which is favorable for transferring surface oxygen to active Cu ion sites. The supported Cu species showed a relatively large particle size as well as less reductive characteristics because the crystallinity of H-CeO_2_ was well developed. The H-Cu-CeO_2_ (800) catalyst showed no α peak and a weak shoulder in both β and γ reduction regions. It shows the major δ reduction peak, which indicated the presence of large crystalline CuO particles [30]. It can be concluded that less developed crystalline CeO_2_ having a large degree of oxygen vacancy helped the supported Cu species in highly dispersed states, while well-developed crystalline CeO_2_ induced the aggregation of Cu species and limited their dispersion. 

**Figure 6:**
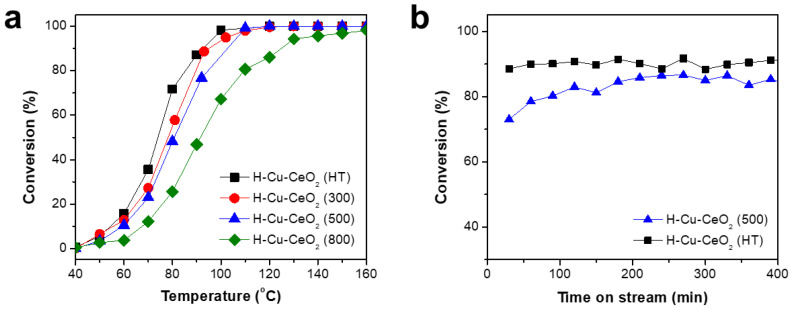
(**a**) CO conversion change as reaction temperature increases and (**b**) time-on-stream test results of the H-Cu-CeO_2_ catalysts.

To evaluate the catalytic activity of the H-Cu-CeO_2_ catalysts, catalytic CO oxidation was carried out using the mixed gas streams of CO (1%), O_2_ (21%), and N_2_ (78%) at varying reaction temperatures. Figure 6a shows the CO conversion change of the H-Cu-CeO_2_ catalysts as the reaction temperature increased. Although the CO conversion value was relatively low, ca. 1% at 40 °C, all the H-Cu-CeO_2_ catalysts exhibited enhanced CO conversion as the reaction temperature increased from 50 to 160 °C. Among the H-Cu-CeO_2_ catalysts employed in this work, the H-Cu-CeO_2_ (HT) catalyst showed the largest CO conversion values at constant temperature. Based on the CO conversion trend at constant temperature, the catalytic activity followed the order: H-Cu-CeO_2_ (HT) > H-Cu-CeO_2_ (300) > H-Cu-CeO_2_ (300) > H-CeO_2_ (800). We compared the T50 and T100 values (temperatures where the CO conversion rates were 50%) of H-Cu-CeO_2_ catalysts for a precise evaluation and summarized. The T50 values were ca. 74, 78, 81, and 91 °C for H-Cu-CeO_2_ (HT), H-Cu-CeO_2_ (300), H-Cu-CeO_2_ (500), and H-CeO_2_ (800), respectively. Based on the above results, Cu supported on H-CeO_2_ having both low support crystallinity and a high degree of oxygen vacancy showed enhanced catalytic activity in CO oxidation. 

To investigate the long-term activity and stability of the H-Cu-CeO_2_ catalysts, the time-on-stream test was carried out at 90 °C for 400 min. Figure 6b shows that both H-Cu-CeO_2_ (HT) and H-Cu-CeO_2_ (500) catalysts revealed a stable activity with negligible deactivation. H-Cu-CeO_2_ (HT) showed higher catalytic performance than H-Cu-CeO_2_ (500). It should be noted that the excellent catalytic performance of the H-Cu-CeO_2_ (HT) catalyst can be mainly attributed to its advantageous characteristics, such as a high degree of oxygen vacancy and favorable metal–support interactions, resulting from a less developed crystallinity of the hollow CeO_2_ support. 

**Figure 7:**
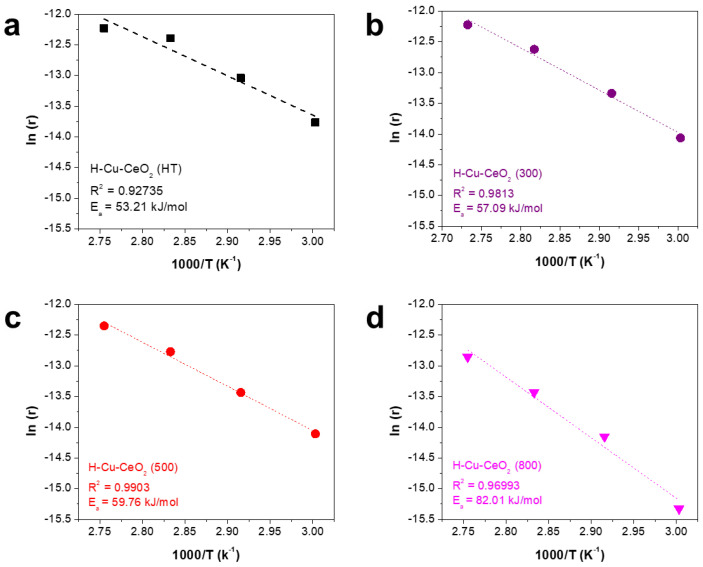
Arrhenius plot for CO oxidation, employing the H-Cu-CeO_2_ catalysts: (**a**) H-CeO_2_ (HT), (**b**) H-CeO_2_ (300), (**c**) H-CeO_2_ (500) and (**d**) H-CeO_2_ (800).

We also estimated the activation energy of the H-Cu-CeO_2_ catalysts at low temperature (range, 60–90 °C) using the Arrhenius plot for CO oxidation. As shown in Figure 7, all H-Cu-CeO_2_ catalysts showed a linear Arrhenius plot between 1/T and ln (r), with a reasonable coefficient of determination (R^2^) in the range of 0.93–0.99. The activation energy values were ca. 53.2, 57.1, 59.76, and 82 kJ/mol for H-Cu-CeO_2_ (HT), H-Cu-CeO_2_ (300), H-Cu-CeO_2_ (500), and H-CeO_2_ (800), respectively, indicating that the H-Cu-CeO_2_ (HT) catalyst was the most active catalyst, and CO oxidation favorably occurred due to the low energy required for the reaction. 

As previously reported and as observed in our study, it should be noted that CO oxidation on H-Cu-CeO_2_ catalysts follows the Mars–van Krevelen redox-type mechanism. Based on the above mechanism, surface oxygen atoms from the CeO_2_ support are essential for the occurring of the oxidation reaction involving adsorbed CO molecules on CuOx clusters as active sites. In our study, a similar mechanism was likely applied during the catalytic CO oxidation using a H-Cu-CeO_2_ catalyst and involved several sequential steps, as follows [29].

(1) Ce^3+^-□-Cu^+^ + CO ↔ Ce^3+^-□-Cu^+^-CO
(2) Ce^3+^- □-Cu^+^-CO + Ce^4+^-O^2-^-Cu^2+^ → 2[Ce^3+^- □-Cu^+^] + CO_2_
(3) 2[Ce^3+^- □-Cu^+^] + O_2_ → 2 [Ce^4+^-O^2-^-Cu^2+^]

First, the CO molecule as a reactant was chemically adsorbed on a Cu^+^ active site, resulting in the formation of a Cu^+^-CO specie. The adsorbed CO molecule migrated to the interface between active CuO and the surface of the CeO_2_ support. CO reacted with the adjacent lattice oxygen provided by the support with the reduction of Ce^4+^ and Cu^2+^. Oxygen vacancies were refilled by the adsorption of oxygen, leading to the oxidation of Ce^3+^ and Cu^+^ to Ce^4+^ and Cu^2+^, respectively [29]. 

It should be noted that the facile migration of surface oxygen atoms of the CeO_2_ support is vital for accelerating CO oxidation. Based on the XRD, Raman analysis, and H_2_-TPR results, CuO-supported hollow CeO_2_ samples having relatively low crystallinity showed a high degree of oxygen vacancy and a large portion of isolated copper oxide species with high dispersion. The highly dispersed Cu species had a higher chance of absorbing CO, and a high degree of oxygen vacancy favored the surface oxygen transfer between closely supported Cu species and the CeO_2_ surface. Therefore, the H-Cu-CeO_2_ (HT) catalyst, which consists of CuO-supported hollow CeO_2_ having less developed CeO_2_ crystallinity, showed a remarkable performance in CO oxidation, with >99% conversion at less than 100 °C.

## 4. Conclusions

We successfully synthesized hollow CeO_2_ nanostructures and controlled their crystallinity. The synthesized CeO_2_ samples were used as support materials for hollow CuO-CeO_2_ catalysts in CO oxidation. The hollow CeO_2_ nanostructures were synthesized by simple hydrothermal strategies, and the crystallinity of the CeO_2_ layer was controlled by varying the calcination temperatures. CeO_2_ crystallinity continuously developed as the calcination temperature increased. As the crystallinity of the hollow CeO_2_ sample varied, the characteristics of the supported Cu species also changed. We observed well-dispersed CuO species and isolated species when CuO was supported on a less-developed crystalline CeO_2_ support having a high degree of oxygen vacancy. However, well-crystallized hollow CeO_2_ supports allowed the Cu species to form large CuO clusters with relatively low metal dispersion. The H-Cu-CeO_2_ (HT) catalyst, which had the lowest CeO_2_ crystallinity, showed a significantly enhanced catalytic activity and catalyst stability in CO oxidation. The remarkable performance indicated that the H-Cu-CeO_2_ (HT) catalyst has not only highly dispersed Cu species for facile CO adsorption but also a high degree of oxygen vacancy, which facilitates the surface oxygen transfer from CeO_2_ to the supported Cu species. We believe that our Cu-supporting hollow CeO_2_ nanostructures described in this study can provide a good and economic solution for developing highly active catalyst materials for various oxidation reactions.

## Supplementary Materials

The following supporting information can be downloaded at: https://www.mdpi.com/article/10.3390/ma15113859/s1, Figure S1: SEM images of H-Cu-CeO_2_ catalysts: (a) H-Cu-CeO_2_ (HT), (b) H-Cu-CeO_2_ (500) and (c) H-Cu-CeO_2_ (800); Figure S2: N_2_ adsorption-desorption isotherms of H-Cu-CeO_2_ catalysts; Figure S3: The XRD patterns of H-Cu-CeO_2_ catalysts in the 2 theta range of 34.5 to 39.5°; Figure S4: XPS results of H-CeO_2_ (x) supports and H-Cu-CeO_2_ (x) catalysts: (a) Ce 3d and (b) Cu 2p spectra; Table S1: Relative mass ratio and estimated CuO loading values of H-Cu-CeO_2_ catalysts.
