# Supplementary material for: Effects of the Crystalline Properties of Hollow Ceria Nanostructures on a CuO-CeO2 Catalyst in CO Oxidation
Source: Materials (Basel). 2022 May 28;15(11):3859. doi: 10.3390/ma15113859 (PMC9181753; doi:10.3390/ma15113859)
Supplement: Supplementary file 1 [file materials-15-03859-s001.zip › materials-1704982-supplementary.pdf]

Supporting Information

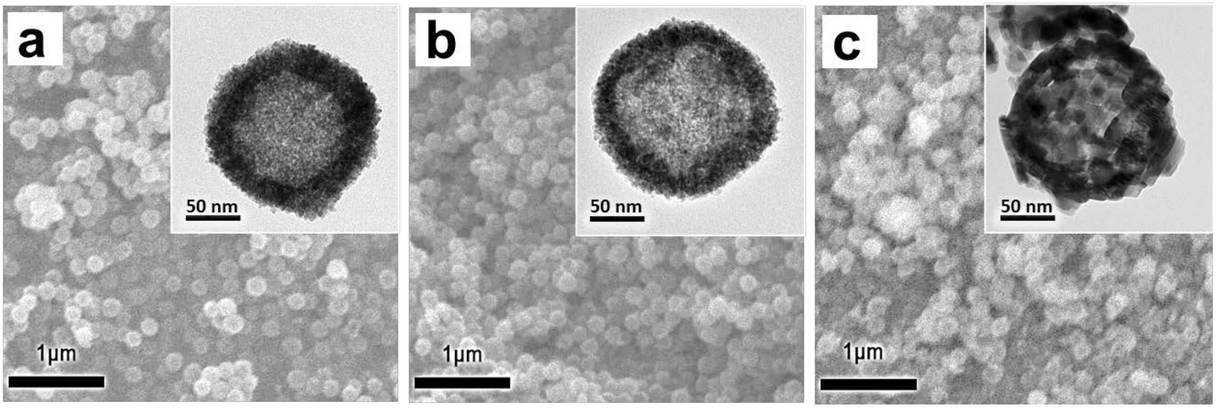

Figure S1. SEM images of H-Cu-CeO_2 catalysts: (a) H-Cu-CeO_2 (HT), (b) H-Cu-CeO_2 (500) and (c) H-Cu-CeO_2 (800)

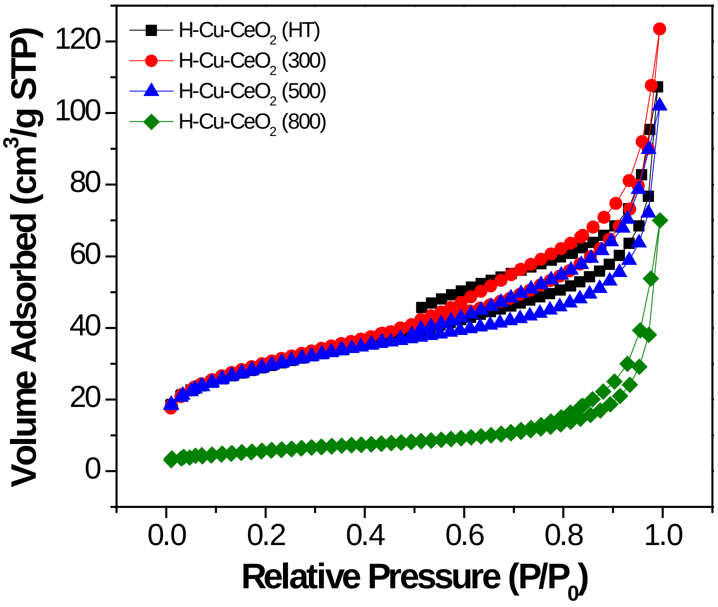

Figure S2. N_2 adsorption-desorption isotherms of H-Cu-CeO_2 catalysts

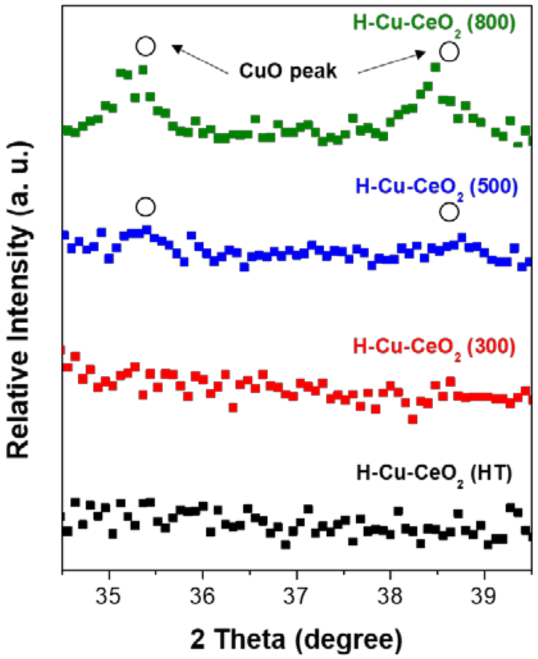

Figure S3. The XRD patterns of H-Cu-CeO_2 catalysts in the 2 theta range of 34.5 to 39.5°

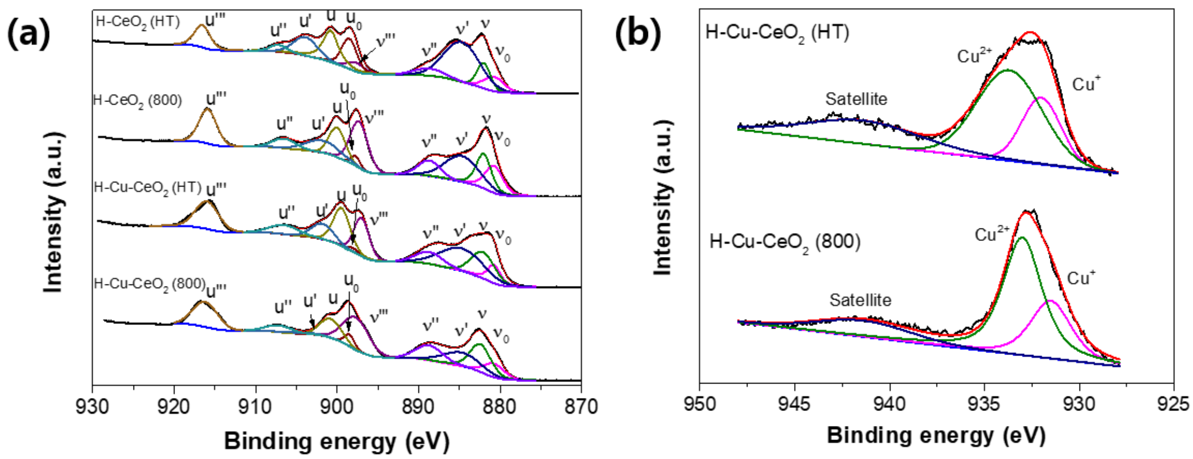

Figure S4. XPS results of H-CeO_2 (x) supports and H-Cu-CeO_2 (x) catalysts: (a) Ce 3d and (b) Cu 2p spectra

Table S1. Relative mass ratio and estimated CuO loading values of H-Cu-CeO_2 catalysts

Sample	Mass ratio			Cu/(Cu+Ce)	Estimated CuO/(CuO+CeO_2)
	Cu	Ce	others		
H-Cu-CeO_2 (HT)	0.1079	0.8724	0.0197	0.1101	0.1114
H-Cu-CeO_2 (500)	0.1135	0.8675	0.0190	0.1157	0.1176
H-Cu-CeO_2 (800)	0.1037	0.8754	0.0209	0.1059	0.1078
